# Alkanethiolate-Capped Palladium Nanoparticles for Regio- and Stereoselective Hydrogenation of Allenes

**DOI:** 10.3390/catal8100428

**Published:** 2018-09-29

**Authors:** Ting-An Chen, Young-Seok Shon

**Affiliations:** Department of Chemistry and Biochemistry, California State University Long Beach, 1250 Bellflower Blvd., Long Beach, CA 90840, USA

**Keywords:** selective hydrogenation, catalysis, nanoparticle, cumulated diene, allene, ligand-capped, semi-heterogeneous

## Abstract

Colloidal Pd nanoparticles capped with octanethiolate ligands have previously shown an excellent selectivity toward the mono-hydrogenation of both isolated and conjugated dienes to internal alkenes. This paper reports an efficient stereoselective mono-hydrogenation of cumulated dienes (allenes) to either Z or E olefinic isomers, depending on the substitution pattern around C=C bonds. Kinetic studies indicate that the reaction progresses through the hydrogenation of less hindered C=C bonds to produce internal Z olefinic isomers. In the cases of di-substitued olefinic products, this initial hydrogenation step is followed by the subsequent isomerization of Z to E isomers. In contrast, the slow isomerization of Z to E isomers for tri-substituted olefinic products results in the preservation of Z stereochemistry. The high selectivity of Pd nanoparticles averting an additional hydrogenation is steered from the controlled electronic and geometric properties of the Pd surface, which are the result of thiolate-induced partial poisoning and surface crowding, respectively. The high activity of colloidal Pd nanoparticle catalysts allows the reactions to be completed at room temperature and atmospheric pressure.

## 1. Introduction

Selective hydrogenation of small alkynes and dienes is an important chemical process because such compounds are reactive monomers for polymerization that decreases the purity of pyrolysis petroleum products [1–3]. Compared to a variety of catalysts investigated for selective hydrogenation of alkynes and conjugated/isolated dienes, only a limited number of studies has been reported for the selective hydrogenation of allenes that have the unique orthogonal π orbital of cumulene. Besides the complete hydrogenation of two π bonds, the partial hydrogenation of allenes can involve either 1,2- or 2,3-hydrogenation. In addition, the products for selective hydrogenation of allenes are often stereoisomers (cis and trans), further complicating the catalysis results. The isomerization of allene groups to alkynes was also observed for several catalytic systems during the hydrogenation of allenes [4].

Earlier studies reported in the literature indicated that the selectivity of supported metal catalysts among different chemo-, regio-, and stereoselective hydrogenation products is generally low for this challenging allene hydrogenation reaction [5–10]. For example, palladium on alumina catalyst, which was synthesized by the hydrothermal method, converted allenes into alkenes, with a relatively low and time-dependent selectivity [9,10]. It has been shown that the pressure of the hydrogen gas would affect the catalytic selectivity of palladium on alumina, which has been correlated to the hydride concentration on the palladium surface. With the lower pressure of H_2_ and at the earlier stage of buta-1,2-diene hydrogenation, *cis*-but-2-ene was the major reaction product. However, with the increased hydrogen pressure above 100 torr or at the later stage of the catalytic reaction, the yields for butane, a full hydrogenation product, would greatly increase with the formation of some *trans*-but-2-ene [10]. During the hydrogenation reaction of buta-1,2-diene, the isomerization products, but-1-yne and buta-1,3-diene, were also observed [9].

**Scheme 1.**
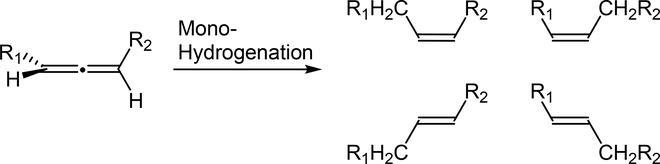
Selective mono-hydrogenation of substituted allenes produces four different stereoisomeric alkenes.

Selective mono-hydrogenation of substituted allenes could produce four different stereoisomeric alkenes depending on the regio- and stereoselective addition of hydrogen, as shown in Scheme 1. Most heterogeneous catalysts, including supported Pd, Pt, Rh, and Ir, produced *cis*-1,2-hydrogenation products as major products, with maximum yields ranging from 70% to 98%, that are highly dependent on the reaction time and hydrogen pressure [5,6,11]. Most of these catalysts produced full hydrogenation products as major products after an extended reaction time. Hydrogenation of allenes with homogeneous catalysts, such as chlorotris(triphenylphosphine) rhodium, allylpalladium (II), and palladium (bis(arylimino)acenaphthene) complexes, also brought about the *cis*-1,2-hydrogenation reaction as its primary pathway [12–14]. With the good selectivity toward the *cis*-1,2-hydrogenation product, the homogeneous palladium (bis(arylimino)acenaphthene) complex was used for the selective hydrogenation of 1,2-allenyl phosphonates to (Z)-1-alkenyl phophonates [14].

With the emergence of the α-amino vinylphosphonate as an important building block for the preparation of biologically active molecules [15], the research on developing heterogeneous catalysts for selective hydrogenation of α-amino allenephosphonates to α-amino vinylphosphonates has drawn interest [16]. The most efficient heterogeneous catalyst for 1,2-hydrogenation was determined to be the Pd/C (10 wt.%) in the presence of quinoline. Depending on the size and functionality of the amino group, however, the stereoselectivity between Z and E isomers varied dramatically, ranging from 58:42 to 96:4, in favor of Z isomers. The yields for 1,2-hydrogenation were as high as 97% for α-amino allenephosphonates, but also as low as 43% for bulkier substrates.

Recently, the first successful and highly efficient 2,3-reduction of substituted allenes was reported by Dong et al. [17]. The Rh-H based homogeneous catalyst with a chiral ligand was able to afford the chemoselective reduction, producing chiral benzylic motifs with good enantioselectivity. The mechanistic studies using isotope labeling revealed that the hydride is added directly to the branched position (3-position) of allenes for 2,3-reduction.

Despite the recent development, a facile method that produces more thermodynamically stable E-alkenes directly from allenes in a single step is currently lacking in the literature. Alkanethiolate-capped palladium nanoparticles (PdNP) have shown a high selectivity for both isolated and conjugated dienes to form mono-hydrogenation products [18–20]. Pd/Al_2_O_3_ coated with alkanethiolate monolayers could selectively hydrogenate unsaturated fatty acids with isolated diene groups to monounsaturated fatty acids [21]. Between two major routes for the conjugated diene hydrogenation, the 1,2- and 1,4-hydrogenation, the thermodynamically stable internal alkenes were produced with high selectivity [20]. This was due to the strong influence of the thiolate ligands on the chemical and electronic properties of the Pd surface. The kinetic study showed that the direct 1,4-addition reaction supplemented by the isomerization of the 1,2-addition products was the main mechanism leading to the internal alkenes in high yields. This article presents the catalytic hydrogenation of cumulated dienes using these PdNPs and the influence of surface capping ligands on their activity and selectivity.

## 2. Results and Discussion

**Scheme 2.**
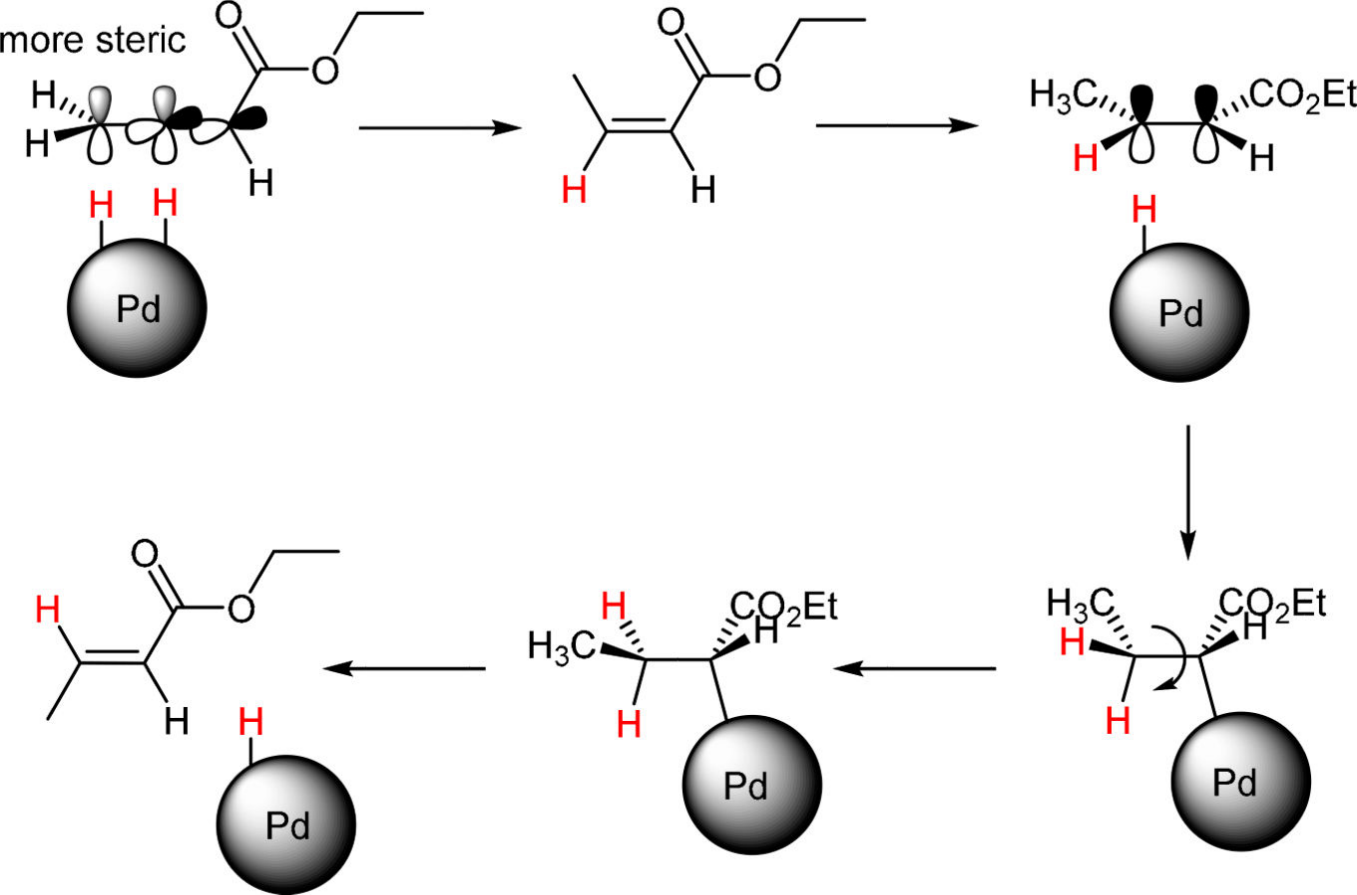
The proposed catalytic reaction mechanism of ethyl buta-2,3-dienoate **1** for the formation of both cis and trans alkenes

The catalytic hydrogenation reaction of ester conjugated cumulene **1** with C8 PdNP was performed at room temperature and atmospheric pressure. The kinetic study of ester conjugated cumulene **1** shown in Table 1 and Figure S1 clearly indicated that the mono-hydrogenated cis-conjugated ester product **2** is the primary hydrogenation product during the early stage of the catalytic hydrogenation. It is reasonable to infer that the steric effect of the substituted ester group was the main cause for the initial preferential formation of cis isomers after mono-hydrogenation, as shown in Scheme 2 [22]. After the 2 h reaction, the yields for the cis product **2** reached over 84%. This result at the early stage of the catalytic reaction was comparable to those of other metal based hydrogenations discussed in the introduction that produce cis products as major products [22,23]. The complete consumption of the substrate **1** required only 3 h, at which the isomerization of the cis isomer **2** to the trans isomer **3** started to take place more rapidly. This suggests that the adsorption of the substrate **1** is more favorable than that of the cis isomer **2**. Once the reactant **1** was consumed, the coordination of the cis isomer **2** followed by the addition of hydrogen produced the mono-σ bonded Pd alkyl intermediate (Scheme 2). After the σ bond rotation to release the steric interaction of the cis isomer, the subsequent α-hydrogen elimination resulted in the formation of the trans isomer **3**, the thermodynamically more stable product. Even with the extended reaction time, a very small amount of the full hydrogenation product, a saturated ester **4** that forms via the di-σ bonded Pd-alkyl intermediate, was generated [24,25]. The isomerized alkyne, ethyl 2-butynoate, was not observed from any of the kinetic study. The carbonyl group was not hydrogenated at all during the C8 PdNP catalysis reaction, indicating a good chemoselectivity toward the C=C bond over the C=O bond hydrogenations [18,19]. The result also showed that the mono-hydrogenation of terminal C=C in the allene group is much more favored over either the mono-hydrogenation of conjugated C=C or the double hydrogenation of both allene C=C bonds. After 24 h of reaction, ester conjugated cumulene **1** was converted to mono-hydrogenated conjugated ester products (~96%, **2** and **3**), with a high selectivity toward trans conjugated ester product **3,** with a yield of ~69%. With an increased reaction time to 36 h, the yields for trans conjugated ester product **3** further increased to ~86%, while the yield for the full hydrogenation product **4** remained mostly the same (from 4% to 6%). The results confirmed a high selectivity for mono-hydrogenation products over full hydrogenation product, with a trans/cis selectivity of up to 10.7.

To investigate the influence of substrate structure on the catalytic activity and selectivity, the catalytic reactions of various allenes using C8 PdNP were attempted (Table 2 and Figures S2–S9). The catalytic hydrogenation of ester conjugated cumulene **5** also produced almost the same results as that of **1**. This indicates that the presence of the phenyl ring in the ester functional group of cumulene **5** would not affect the catalytic reactivity of C8 PdNP. The presence of the methyl group at the C4 position of internal cumulated diene **9** seemed to enhance the cis to trans isomerization. Hence, the catalytic reaction of **9** produced trans conjugated ester **11** as the major product without yielding cis conjugated ester **10** after the extended equilibrium between two conjugated stereoisomers. However, the further reduction of internal cumulated diene **9** to fully hydrogenated ester seemed to be also facilitated based on the increased yield of saturated ester **12**. This is because the cis to trans isomerization of isomer **10** with a large ethyl substituent could be sterically more inhibited, facilitating the addition of second hydrogen to the mono-σ-bonded Pd-alkyl intermediate formed after the addition of the first hydrogen to the adsorbed C=C bond of the cis isomer **10**. These three results indicated that C8 PdNP promotes the selective hydrogenation of conjugated allenes to thermodynamically stable trans-isomers with good stereo- and regioselectivity.

For the reaction of ester conjugated cumulene **13**, the methyl group on C2 (αC from ester group) of the substituted cumulene eliminated the full hydrogenation of the allene group, resulting in the quantitative formation of the mono-hydrogenated Z and E conjugated ester products, **14** and **15**. However, the selectivity between Z and E isomers was reversed for the reaction of ester conjugated cumulene **13** in comparison to those of two less sterically hindered substrates, **1** and **5,** and also di-substituted internal cumulene **9**. After the formation of kinetically favored Z isomer **14**, the isomerization of Z to E isomer (**15**) requires the adsorption of Z isomer and the Pd-H addition to the more sterically bulky C=C bond. It appears that the substituted methyl group at the C2 position hindered this process and slowed the isomerization down, producing the Z isomer **14** as the major product even after the prolonged reaction time. For the catalytic hydrogenation of the diester substrate **17**, the kinetically favored product, the Z isomer **18** was again the major product, with a selectivity over 90%. The adsorption and hydrogen addition occurs via the less sterically hindered coordination complex. The presence of large ester groups prevented the additional adsorption and hydrogen addition, inhibiting both the isomerization of Z isomer **18** to E isomer **19** and the second hydrogenation to the saturated ester product **20**. The extended reaction time did not change the selectivity for the final mono-hydrogenated conjugated ester products, **18** and **19**. The overall results clearly demonstrated that C8 PdNP synthesized using the thiosulfate protocol has the unique catalytic property to form the Z mono-hydrogenated conjugated esters from ester conjugated cumulenes in the early stage of reactions and then further isomerize the Z alkene to the E alkene of conjugated esters depending on the structure of the substrate, especially the degree of substitution at the C2 position (mono-substituted at C2: E alkene, di-substituted at C2: Z alkene).

To examine the role of the conjugated ester group during the catalytic reaction of allenes, the kinetic investigation of the hydrogenation of 1-cyclohexylpropa-1,2-diene **21** was performed (Table 3, Figure S10). The results showed that the reactant consumption of **21** is slower than that of the ester conjugated cumulene **1**. In addition, the hydrogenation selectivity for the terminal C=C bond of the allene group was much lower at the completion of substrate consumption (100% for **1** at 3 h vs. 72% (Z + E) for **21** at 4 h). Significant hydrogenation of the internal C=C bond of allene **21** took place due to the absence of conjugated ester that stabilizes the internal C=C bond. With te enhanced reactivity of the internal C=C bond, the catalytic reaction of 1-cyclohexylpropa-1,2-diene **21** produced 28% of the terminal alkene **24** after 4 h of reaction. As seen from the reactions of all ester conjugated cumulenes, the cis alkene **22** was the major product at the early stage of the hydrogenation reaction for 1-cyclohexylpropa-1,2-diene **21**. With the increased reaction time, the cis internal alkene **22** was isomerized to the trans internal alkene **23**. The terminal alkene **24** was also converted to the internal alkene by isomerization, which was accompanied by the formation of the full hydrogenation product **25** as a minor product.

Considering the interesting catalytic property of C8 PdNP for ester conjugated cumulenes, the catalytic hydrogenation of some other unsaturated substrates that would generate same conjugated ester as the major product was compared. In addition, dual-substrates catalytic reactions were also attempted (Table 4 and Figures S11–S14). For the catalytic reaction of ethyl but-3-enoate **26**, ethyl *trans*-but-2-enoate **3**, the trans conjugated ester, was obtained as the major product after the isomerization of the terminal C=C bond to the internal C=C bond. The greater selectivity for the isomerization over the hydrogenation indicated that the reaction mostly involves the mono-σ bonded branched Pd alkyl intermediate. In comparison, the reaction of **1** that produced the cis isomer as the initial major product would take place via the di-σ bonded Pd alkyl intermediate. The hydrogenation of the substrate, **26**, via the di-σ bonded Pd alkyl intermediate was only limited to ~17%, which was resulted from the impediment of ethyl but-3-enoate **26** in establishing the strong π bond coordination on to the poisoned Pd surface. On the other hand, the catalytic reaction of ethyl but-2-ynoate **27** mostly produced the mono-hydrogenation products, with ethyl *cis*-but-2-enoate **2** as the major product, after 24 h. It showed that the hydrogenation reaction via the di-σ bonded Pd-alkyl intermediate was the major route producing the cis-product. The small amount of remaining substrate after a 24 h reaction indicated that this hydrogenation of ethyl but-2-ynoate **27** is kinetically slower than the reactions of **1** and **26**. When the reaction was continued beyond 24 h, the subsequent isomerization of the cis conjugated ester **2** to the trans conjugated ester **3** took place as indicated by the results analyzed after 48 h of reaction. The presence of the unreacted alkyne substrate, **27,** seems to slow down the isomerization of the cis conjugated ester **2** to the trans conjugated ester **3**. The dual-substrates catalytic reactions comparing the combinations of **1** and **26** vs. **1** and **27** provided additional evidence supporting the slow hydrogenation activity of ethyl but-2-ynoate **27**. The presence of alkyne substrate also inhibited or slowed the subsequent isomerization of the cis conjugated ester **2** to the trans conjugated ester **3**. All these results indicated that C8 PdNP has a highly predictable selectivity to form different isomers based on the structure of substrates and the formation of different reaction intermediates as the results of both surface crowding and partial poisoning effects [26–28].

## 3. Materials and Methods

### 3.1. Materials

All reagents were purchased from the following suppliers and used as received without further purification. 1-Bromooctane (C_8_H_17_Br), sodium thiosulfate pentahydrate (Na_2_O_3_S_2_·5H_2_O), and substrates (ethyl buta-2,3-dienoate, benzyl buta-2,3-dienoate, ethyl-2-methyl buta-2,3-dienoate, diethyl buta-1,2-dieno-3,4-dicarboxylate, ethyl penta-2,3-dienoate, and ethyl but-2-ynoate) were obtained from Sigma-Aldrich (St. Louis, MO, USA). Tetra-*n*-octylammonium bromide (TOAB), sodium borohydride (NaBH_4_), and potassium tetrachloropalladate(II) (K_2_PdCl_4_) were obtained from Acros (Hampton, NH, USA). Ethanol, methanol, acetone, and toluene were obtained from Fisher Scientific (Hampton, NH, USA). Chloroform-d (CDCl_3_) was purchased from Cambridge Isotope Laboratories, Inc. (Tewksbury, MA, USA) Water was purified by Barnstead NANO pure Diamond ion exchange resins purification unit.

### 3.2. Synthesis of Sodium S-Octylthiosulfate

Sodium thiosulfate pentahydrate (20 mmol) and 1-bromooctane (20 mmol) were mixed in the mixture of water and ethanol (1/1 volume ratio) solution and refluxed for 3 h. The crude product was further recrystallized using hot ethanol. ^1^H NMR (400 MHz, D_2_O): *δ* 3.1 ppm (triplet, α-CH_2_-S), *δ* 1.7 ppm (quintet, β-CH_2_CH_2_-S), *δ* 1.3 ppm (broad peak, -CH_2_-), and *δ* 0.90 ppm (triplet, CH_3_-). More characterization results are available in the previous publication [20].

### 3.3. Synthesis of Octanethiolate-Capped Palladium Nanoparticles (C8 PdNP)

K_2_PdCl_4_ (0.4 mmol) and sodium *S*-octylthiosulfate ligand (0.8 mmol) were transferred from aqueous phase to organic phase by a phase transfer agent of TOAB (4 mmol) and then reduced by NaBH_4_ (8 mmol). Ethanol and methanol were used to wash the excess TOAB after reaction was completed. Nanoparticle size was estimated to be 2.3 ± 1.2 nm. The organic weight fraction of 19% and palladium weight fraction of 81% based on the thermogravimetric analysis. These characterization results are available in the previous publication [20].

### 3.4. Synthesis of Ethyl but-3-enoate (Compound 26)

Ethyl but-3-enolate was synthesized following the published procedure [29]. Briefly, pentane (5 mL) was placed in a 50 mL round bottle flask with ethanol (863 μL) and trimethylamine (1.34 mL) under 0 °C. Crotonoyl chloride (916 μL) was dissolved in pentane (5 mL) and the solution was added dropwise into the round bottle flask for 10 min. The solution was then removed from an ice bath and stirred for 3 h at room temperature. Saturated NaHCO_3_ (1 mL) and water (10 mL) were added in the round bottle flask after the 3 h reaction. Diethyl ether (12 mL) was added and the reaction mixture was extracted using a separatory funnel. The organic solution was further dried by sodium sulfate before the removal of solvent using rotary evaporation. The purity and structure of the obtained product were confirmed according to the characterization data in the literature [29].

### 3.5. Catalysis Experiments

The substrates (0.5 mmol) with 5 mol % of octanethiolate-capped palladium nanoparticle (Pd: 0.025 mmol) were mixed in a 2.5 mL CDCl_3_ in the 50 mL round bottom flask and purged with H_2_ for 10 min. For 36 and 48 h experiments, the second 5 min H_2_ purge would be needed after 24 h of the first purge. The composition of the crude product containing octanethiolate-capped palladium nanoparticle (C8 PdNP) were characterized by Bruker Fourier 400 MHz NMR.

## 4. Conclusions

The catalytic reactions of various unsaturated organic substrates using octanethiolate-capped Pd nanoparticle demonstrated that this catalyst has an excellent potential for the selective hydrogenation of the less hindered C=C bond of cumulated dienes conjugated with an ester group. Initially, the mono-hydrogenation produced the cis (or Z) conjugated esters with a high selectivity. The subsequent isomerization of the cis conjugated esters to the trans conjugated esters took place for the compounds that possess no steric group at C2 carbon of conjugated esters. For the substrates containing a steric group, the Z product would remain as the major product. This interesting activity and selectivity of the catalyst is due to the partial poisoning and surface crowding effects of octanethiolate ligands on the Pd nanoparticle surface. The hydrogenation of the second C=C bond is inhibited without the presence of an additional π coordinating group that enhances the adsorption of the substrate. With a decreased activity for hydrogenation, the alkanethiolate-capped Pd nanoparticle catalyst would isomerize the terminal C=C bond to an internal C=C bond and cis alkene to trans alkene. This study clearly demonstrates the strong influence of alkanethiolate passivation on the electronic and geometric properties of colloidal nanoparticle catalysts.

## Supplementary Material

Supplemental

## Figures and Tables

**Table 1.** Catalytic reaction of ethyl buta-2,3-dienoate 1 ^a^.

Substrate	Product Composition			
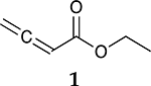				
**Reaction Time**	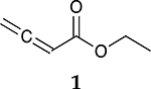	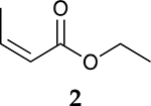	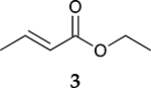	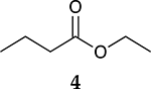

0.5 h	86%	13%	1%	0%
1 h	71%	26%	3%	0%
2 h	10%	84%	6%	0%
3 h	0%	76%	24%	0%
4 h	0%	67%	30%	3%
24 h	0%	27%	69%	4%
36 h	0%	8%	86%	6%

a Reaction condition: 0.5 mmol substrate, 5 mol % Pd catalyst (0.025 mmol Pd), 2.5 mL CDCl_3_, 1 atm H_2_.

**Table 2.** Catalytic reaction of conjugated ester functionalized allenes ^a^.

Substrate Reaction Time	Product Composition			Conv.
	cis or Z	trans or E	Full Hydrogenation	
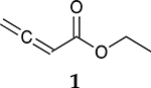	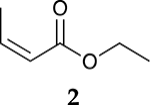	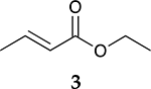	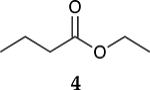	
24 h	27%	69%	4%	100%
36 h	8%	86%	6%	100%

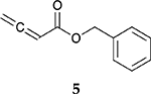	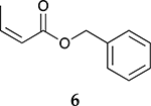	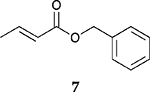	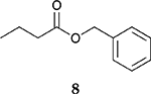	
24 h	26%	70%	4%	100%
36 h	14%	82%	4%	100%

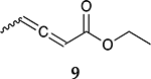	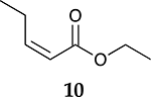	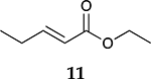	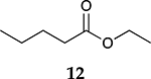	
24 h	20%	71%	9%	100%
48 h	0%	82%	18%	100%

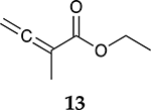	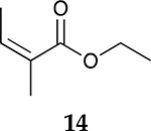	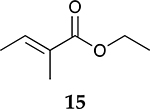	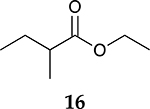	
24 h	86%	14%	0%	100%
36 h	73%	27%	0%	100%

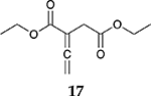	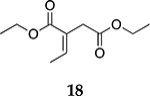	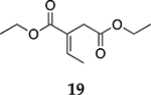	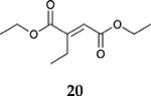	
24 h & 48 h	90%	10%	0%	100%

a Reaction condition, 0.5 mmol substrate, 5 mol % Pd catalyst (0.025 mmol Pd), 2.5 mL CDCl_3_, 1 atm H_2_.

**Table 3.** Kinetic study of the catalytic reaction of 1-cyclohexylpropa-1,2-diene 21 ^a^.

Substrate	Product Composition				
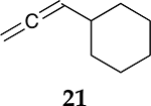					
**Reaction Time**	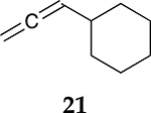	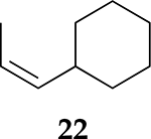	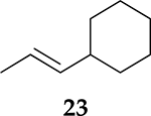	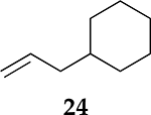	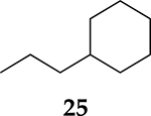

2 h	42%	36%	7%	17%	0%
4 h	0%	63%	9%	28%	0%
24 h	0%	37%	52%	0%	11%

a Reaction condition,0.5 mmol cumulene, 5 mol % Pd catalyst (0.025 mmol Pd), 2.5 mL CDCl_3_, 1 atm H_2_.

**Table 4.** The single-substrate and dual-substrates catalytic reaction of various unsaturated esters.

	Reactant		Product		
Trial 1		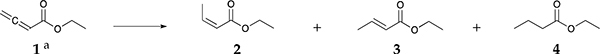			
	
		0%	27%	69%	4%

Trial 2		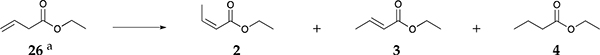			
	
		0%	7%	76%	17%

Trial 3		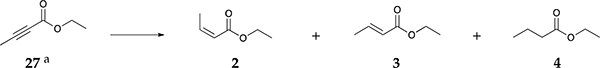			
	
		3%	86%	10%	1%
		0% ^b^	23% ^b^	73% ^b^	4% ^b^

Trial 4	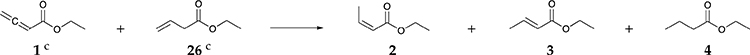				
	
	0%	0%	22%	67%	11%

Trial 5	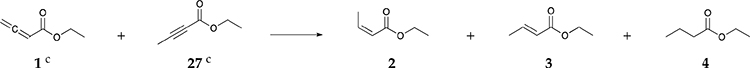				
	
	0%	0%	66%	32%	2%

a 0.5 mmol substrate, 5 mol % Pd catalyst (0.025 mmol Pd), 2.5 mL CDCl_3_, 1 atm H_2_, 24 h reaction

b 48 h reaction time

c Each individual substrate is 0.25 mmol, 5 mol % Pd catalyst (0.0125 mmol), 2.5 mL CDCl_3_, 1 atm H_2_, 24 h reaction.
