# Supplementary material for: Alkanethiolate-Capped Palladium Nanoparticles for Regio- and Stereoselective Hydrogenation of Allenes
Source: Catalysts. Author manuscript; Available in PMC 2019 Feb 5. (PMC6363366; doi:10.3390/catal8100428)
Supplement: Supplemental [file NIHMS1005658-supplement-Supplemental.pdf]

Supporting information

Alkanethiolate-Capped Palladium Nanoparticles for Regio- and Stereoselective Hydrogenation of Allenes

Ting-An Chen and Young-Seok Shon*

Department of Chemistry and Biochemistry, California State University, Long Beach. 1250 Bellflower Blvd. Long Beach, CA 90840. *Email: ys.shon@csulb.edu.

Figure S1. The ^{1}H NMR kinetic study of compound **1** with 5 mol% of C8 PdNP under 1 atm H_2 in $CDCl_3$. The integrations only represent for 24th hour reaction. S-2
Figure S2. The ^{1}H NMR of compound **1** after the 36 hour reaction with 5 mol% of C8 PdNP under 1 atm H_2 in $CDCl_3$. S-3
Figure S3. The ^{1}H NMR of compound **5** after the 24 hour reaction with 5 mol% of C8 PdNP under 1 atm H_2 in $CDCl_3$. S-4
Figure S4. The ^{1}H NMR of compound **5** after the 36 hour reaction with 5 mol% of C8 PdNP under 1 atm H_2 in $CDCl_3$. S-5
Figure S5. The ^{1}H NMR of compound **9** after the 24 hour reaction with 5 mol% of C8 PdNP under 1 atm H_2 in $CDCl_3$. S-6
Figure S6. The ^{1}H NMR of compound **9** after the 48 hour reaction with 5 mol% of C8 PdNP under 1 atm H_2 in $CDCl_3$. S-7
Figure S7. The ^{1}H NMR of compound **13** after the 24 hour reaction with 5 mol% of C8 PdNP under 1 atm H_2 in $CDCl_3$. S-8
Figure S8. The ^{1}H NMR of compound **13** after the 36 hour reaction with 5 mol% of C8 PdNP under 1 atm H_2 in $CDCl_3$. S-9
Figure S9. The ^{1}H NMR of compound **17** (top) and after the 24 hour reaction (bottom) with 5 mol% of C8 PdNP under 1 atm H_2 in $CDCl_3$. S-10
Figure S10. The ^{1}H NMR of compound **21** (top), after the 2 hour reaction (second), after the 4 hour reaction (third), and after the 24 hour reaction (bottom) with 5 mol% of C8 PdNP under 1 atm H_2 in $CDCl_3$. S-11
Figure S11. The ^{1}H NMR of compound **26** (top) and after the 24 hour reaction (bottom) with 5 mol% of C8 PdNP under 1 atm H_2 in $CDCl_3$. S-12
Figure S12. The ^{1}H NMR of compound **27** (top), after the 24 hour reaction (middle), and after the 48 hour reaction (bottom) with 5 mol% of C8 PdNP under 1 atm H_2 in $CDCl_3$. S-13
Figure S13. ^{1}H NMR of compound **1** and **26** dual-substrates after the 24 hour reaction with 5 mol% of C8 PdNP under 1 atm H_2 in $CDCl_3$. S-14
Figure S14. The ^{1}H NMR of compound **1** and **27** dual-substrates after the 24 hour reaction with 5 mol% of C8 PdNP under 1 atm H_2 in $CDCl_3$. S-15

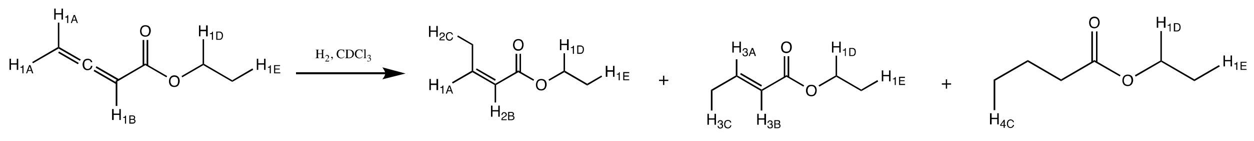

1 *2* *3* *4*

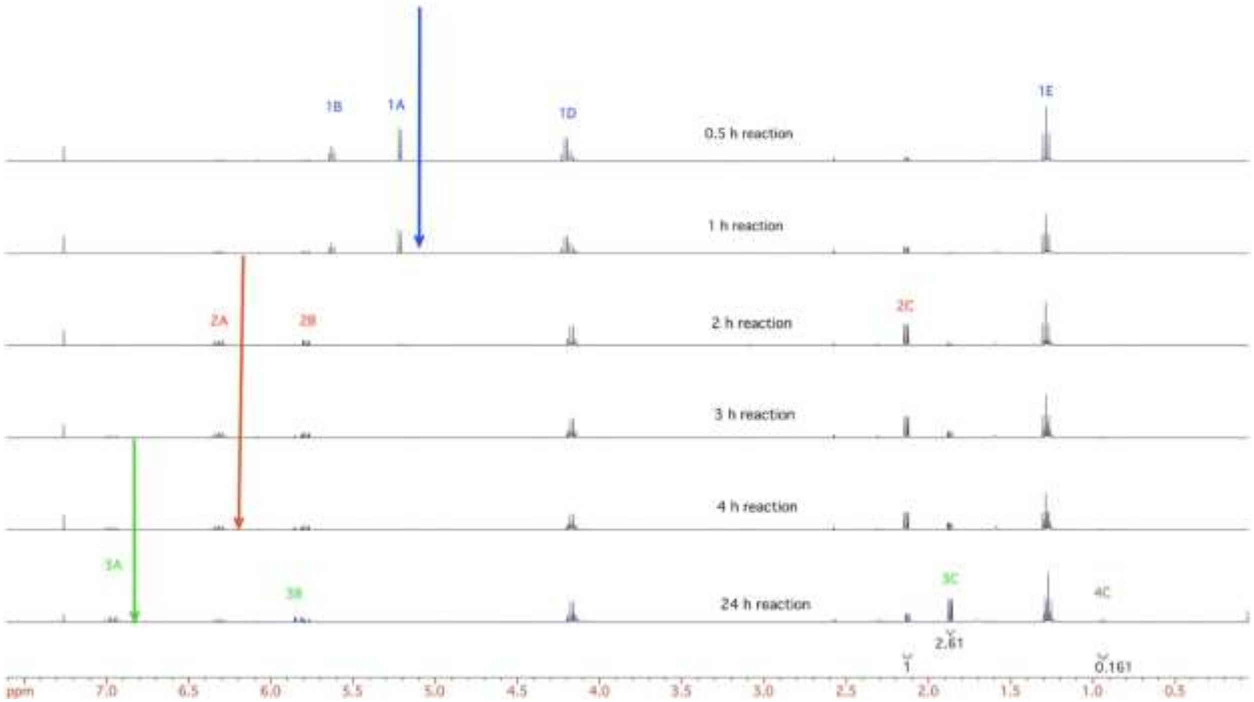

Figure S1. The ^{1}H NMR kinetic study of compound **1** with 5 mol% of C8 PdNP under 1 atm H_2 in $CDCl_3$. The integrations only represent for 24th hour reaction.

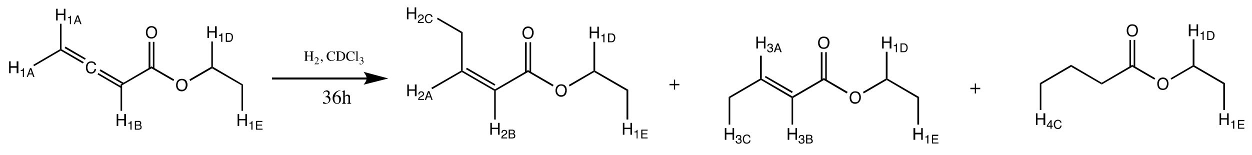

1 *2* *3* *4*

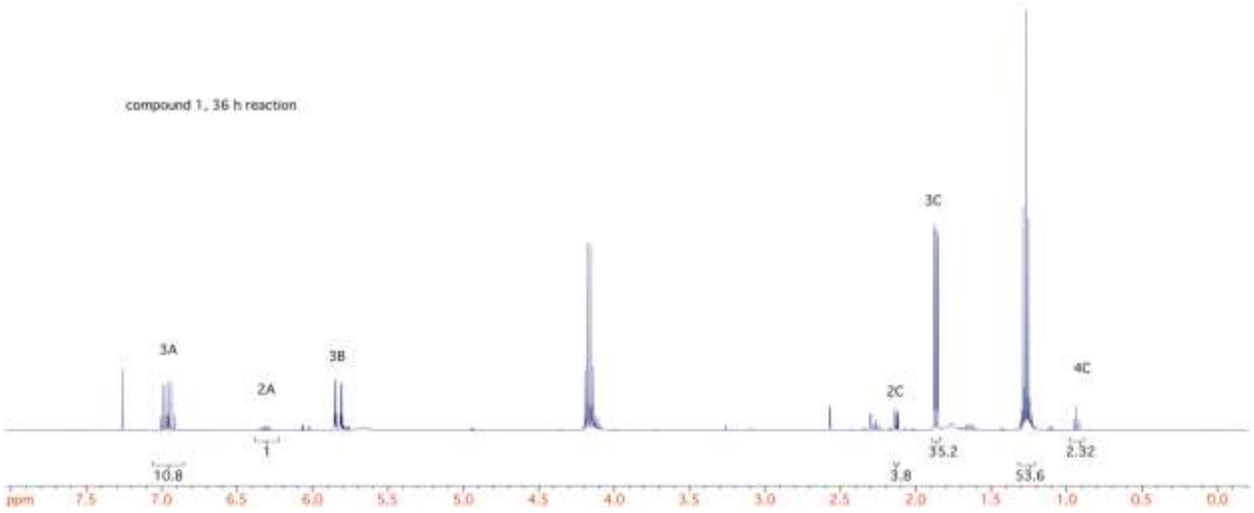

Figure S2. The ^{1}H NMR of compound **1** after the 36 hour reaction with 5 mol% of C8 PdNP under 1 atm H_2 in $CDCl_3$.

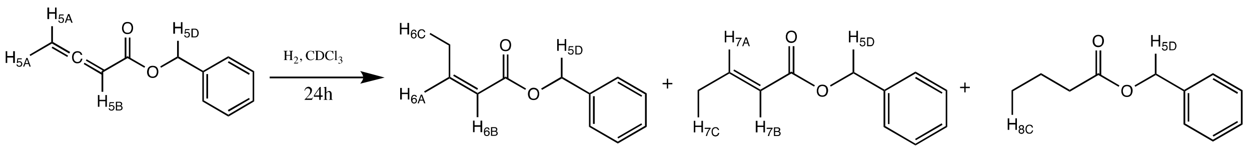

5 6 7 8

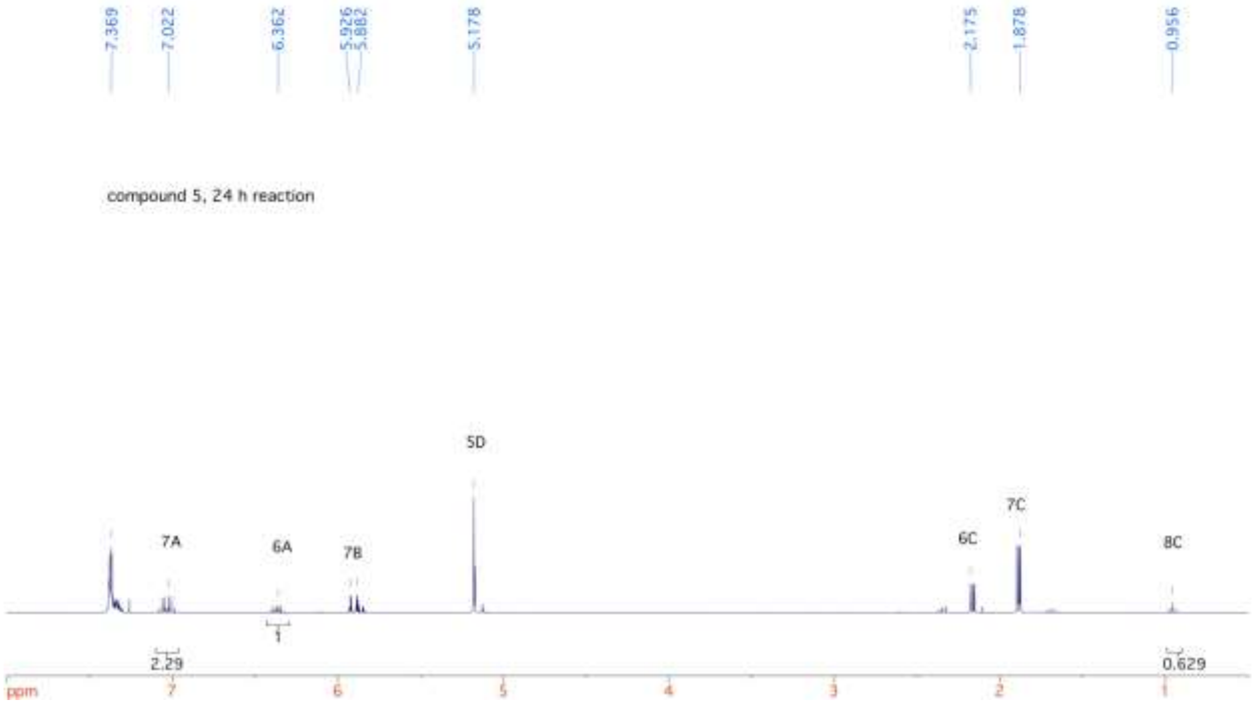

Figure S3. The ^{1}H NMR of compound **5** after the 24 hour reaction with 5 mol% of C8 PdNP under 1 atm H_2 in $CDCl_3$.

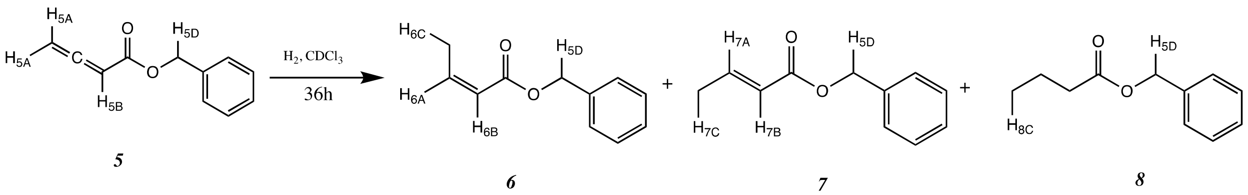

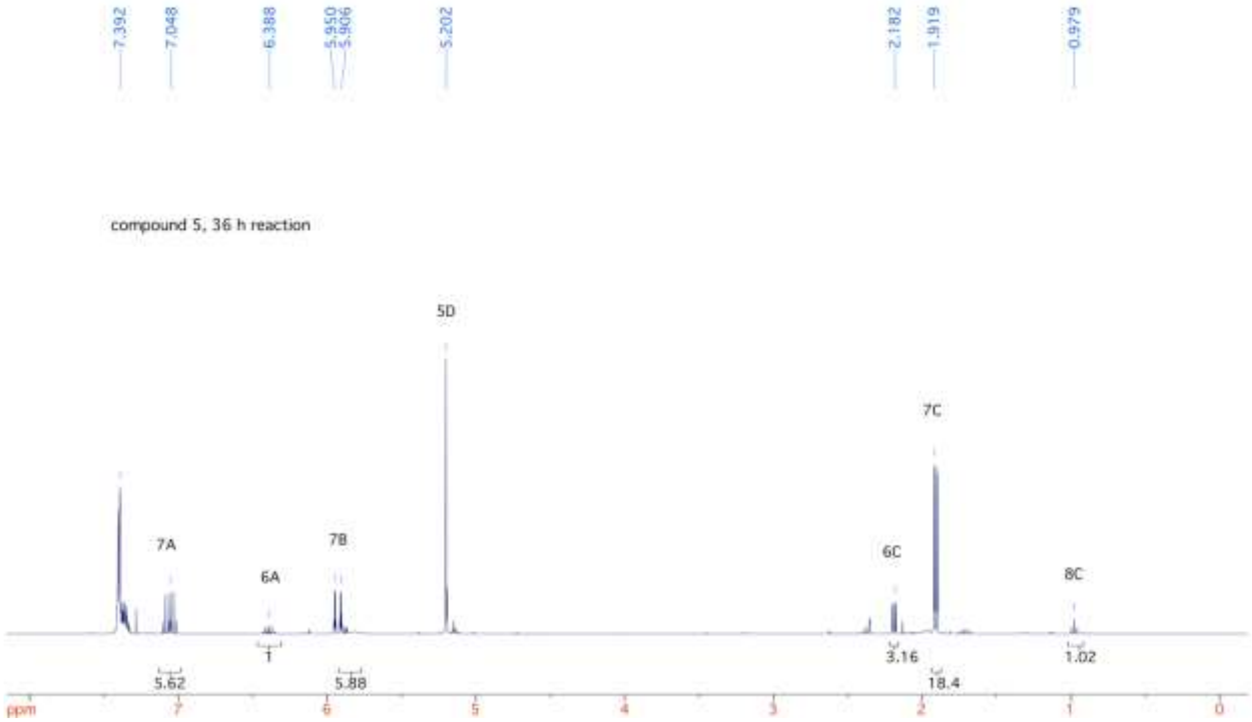

Figure S4. The ^{1}H NMR of compound **5** after the 36 hour reaction with 5 mol% of C8 PdNP under 1 atm H_2 in $CDCl_3$.

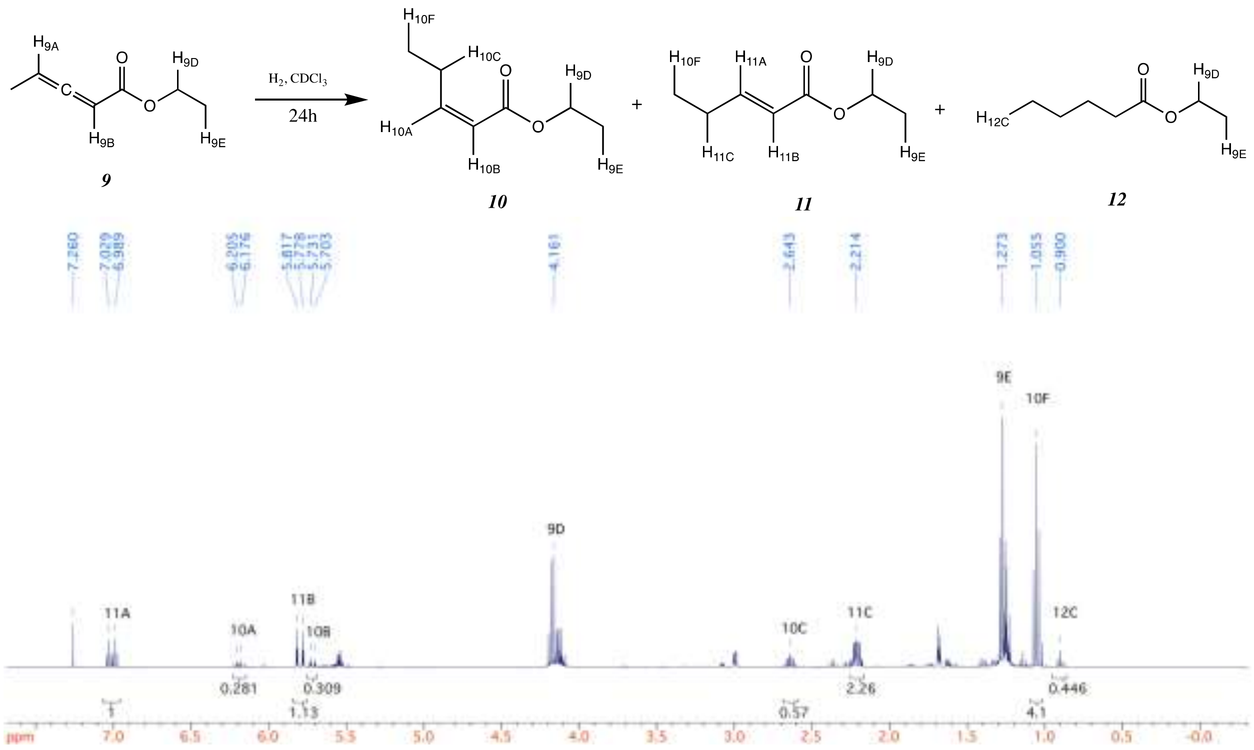

Figure S5. The ^{1}H NMR of compound **9** after the 24 hour reaction with 5 mol% of C8 PdNP under 1 atm H_2 in $CDCl_3$.

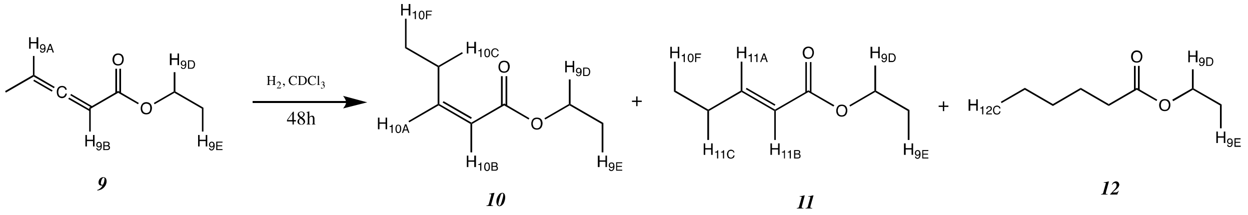

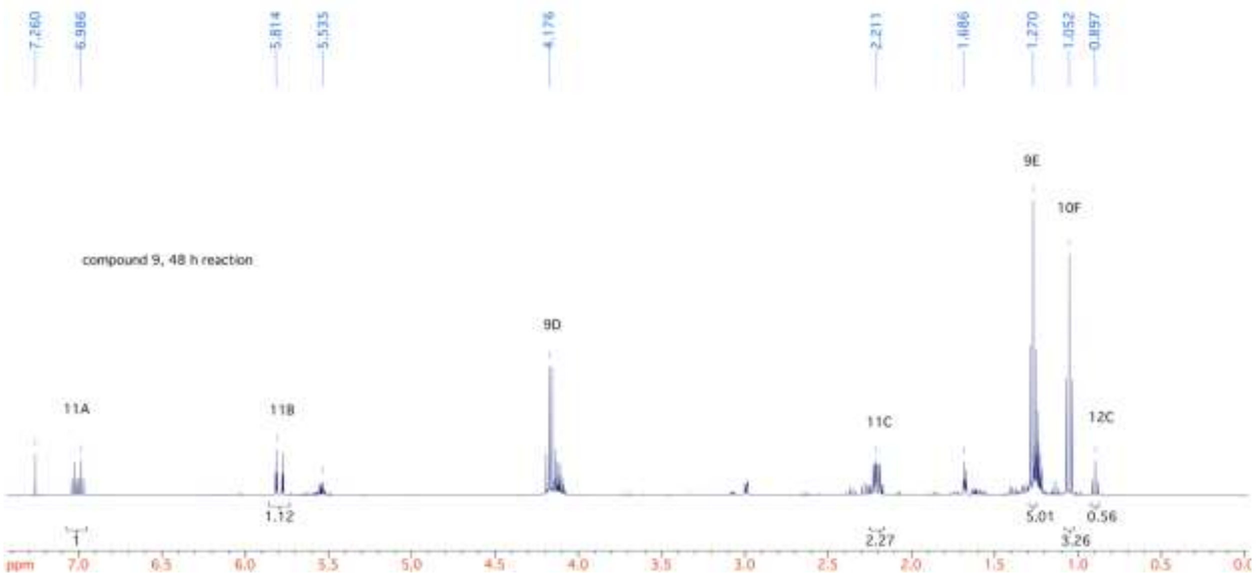

Figure S6. The ^{1}H NMR of compound **9** after the 48 hour reaction with 5 mol% of C8 PdNP under 1 atm H_2 in $CDCl_3$.

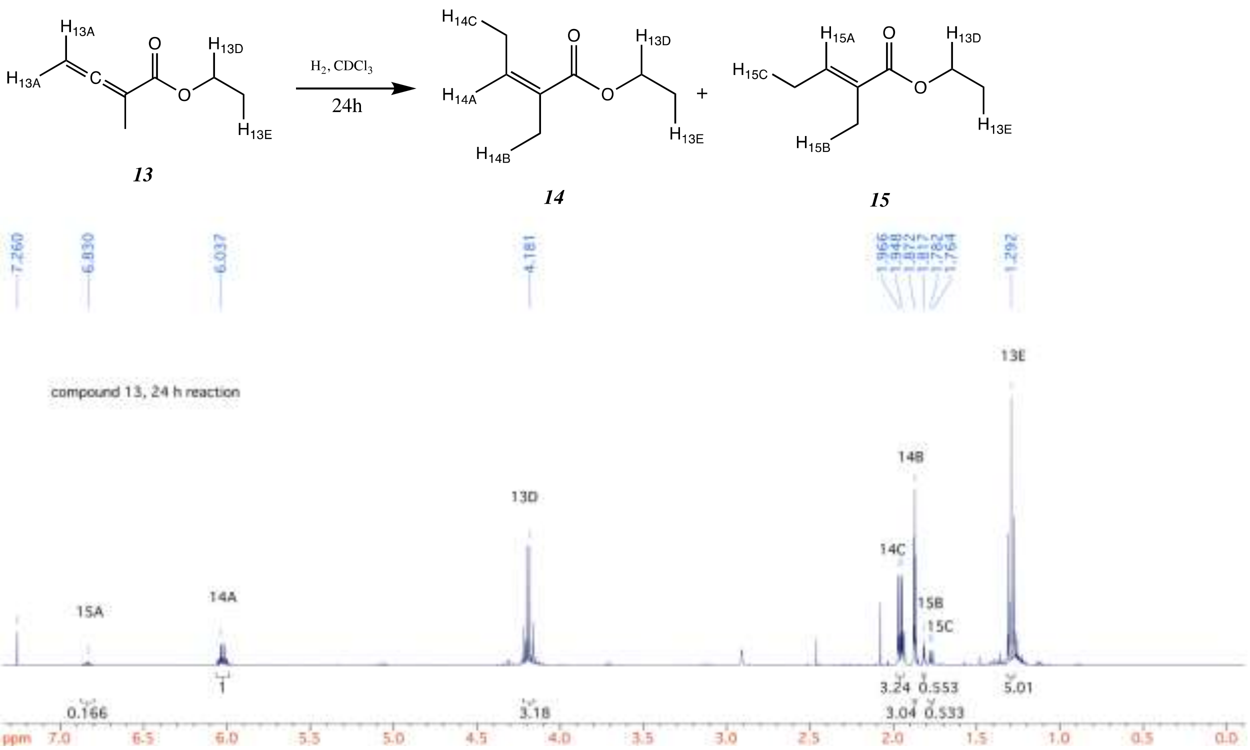

Figure S7. The ^{1}H NMR of compound **13** after the 24 hour reaction with 5 mol% of C8 PdNP under 1 atm H_2 in $CDCl_3$.

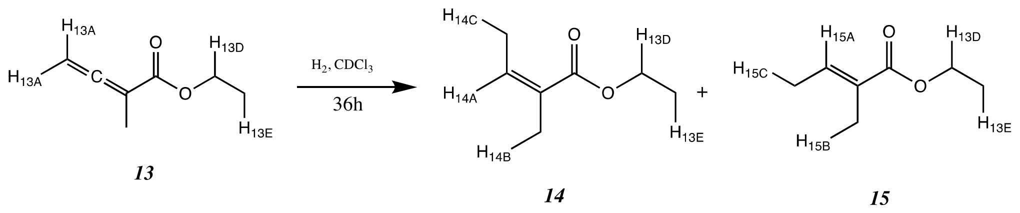

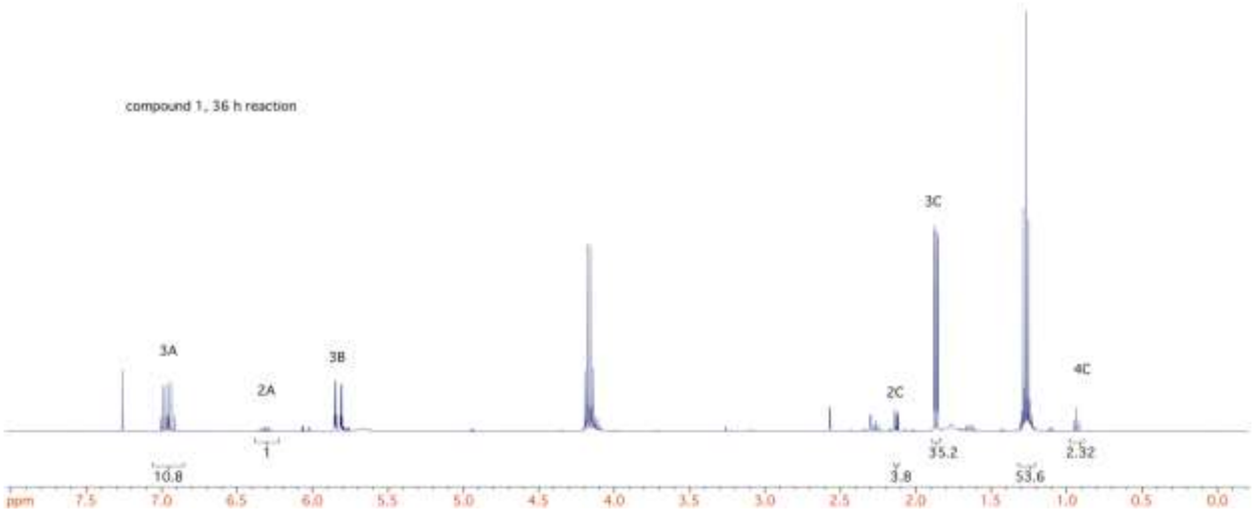

Figure S8. The ^{1}H NMR of compound **13** after the 36 hour reaction with 5 mol% of C8 PdNP under 1 atm H_2 in $CDCl_3$.

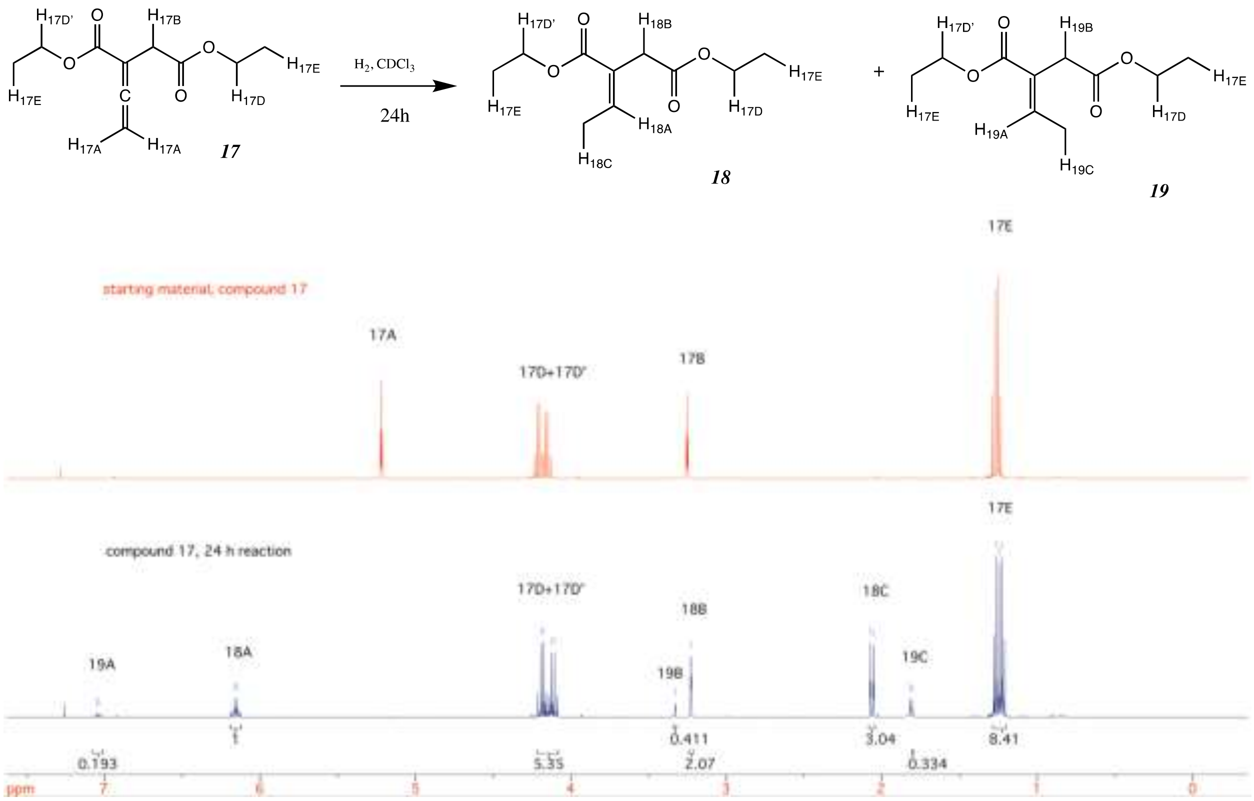

Figure S9. The ^{1}H NMR of compound **17** (top) and after the 24 hour reaction (bottom) with 5 mol% of C8 PdNP under 1 atm H_2 in $CDCl_3$.

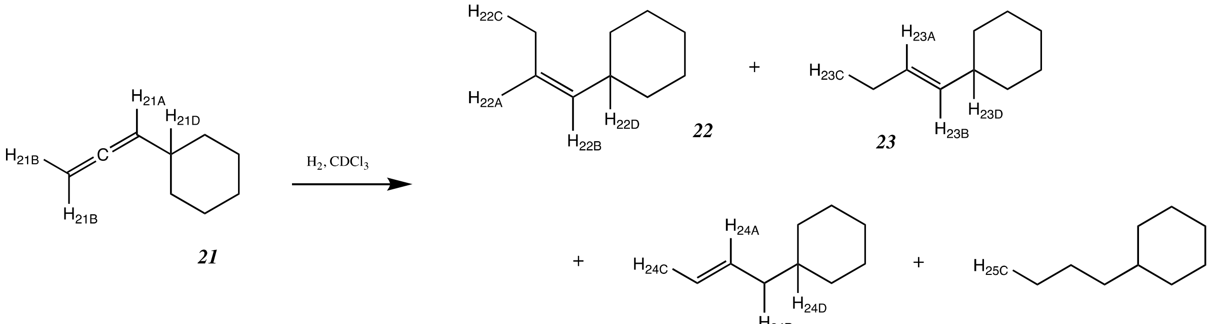

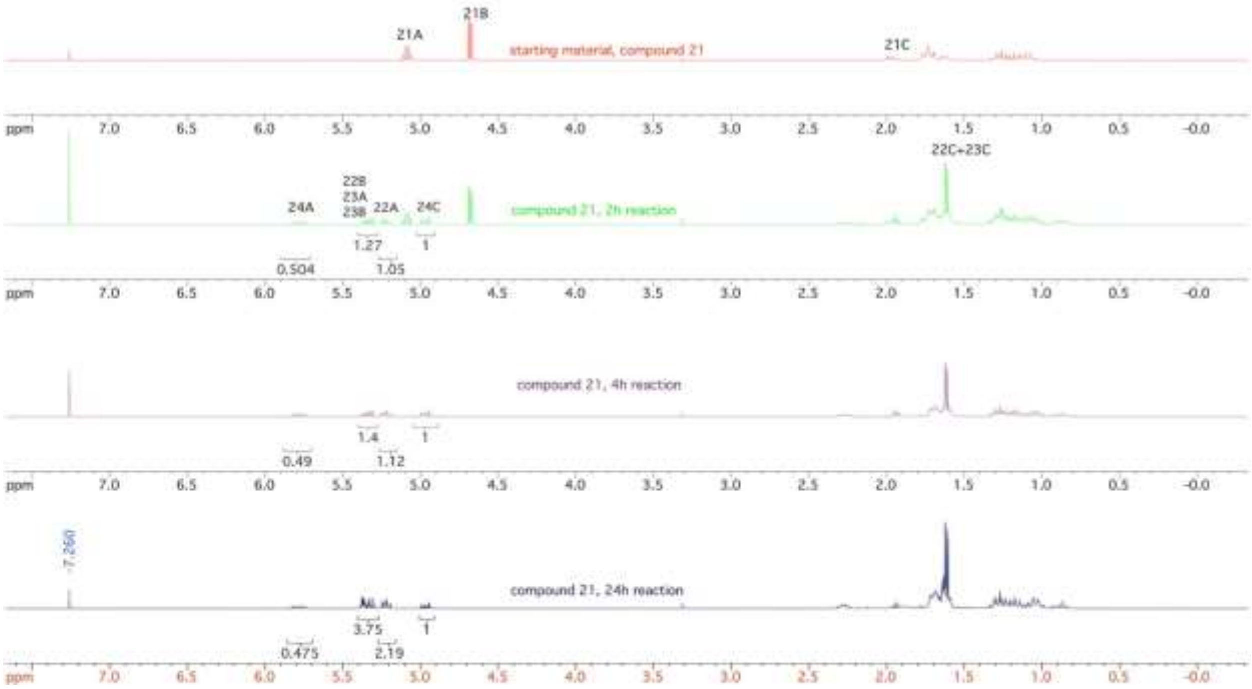

Figure S10. The ^{1}H NMR of compound **21** (top), after the 2 hour reaction (second), after the 4 hour reaction (third), and after the 24 hour reaction (bottom) with 5 mol% of C8 PdNP under 1 atm H_2 in $CDCl_3$.

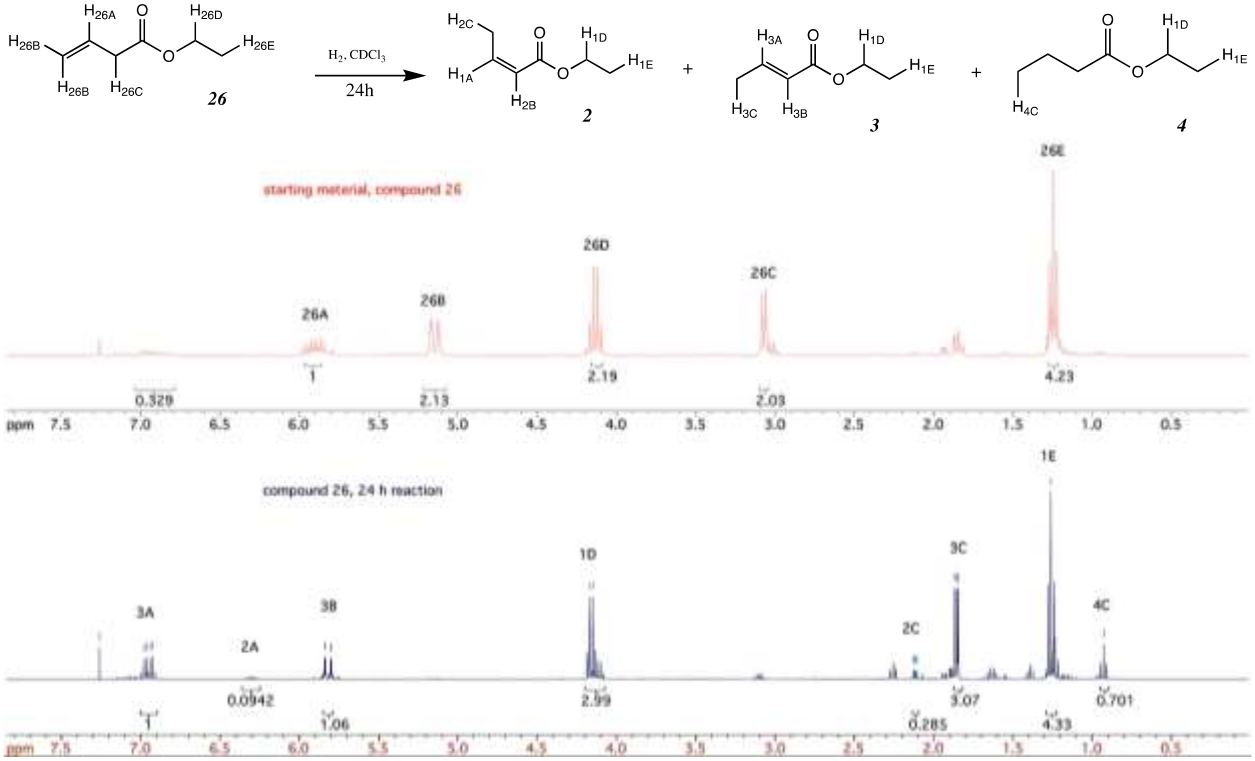

Figure S11. The ^{1}H NMR of compound **26** (top) and after the 24 hour reaction (bottom) with 5 mol% of C8 PdNP under 1 atm H_2 in $CDCl_3$.

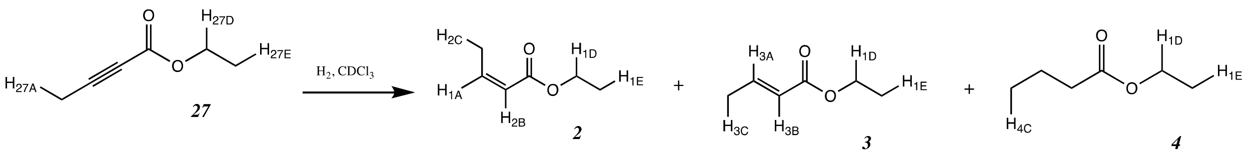

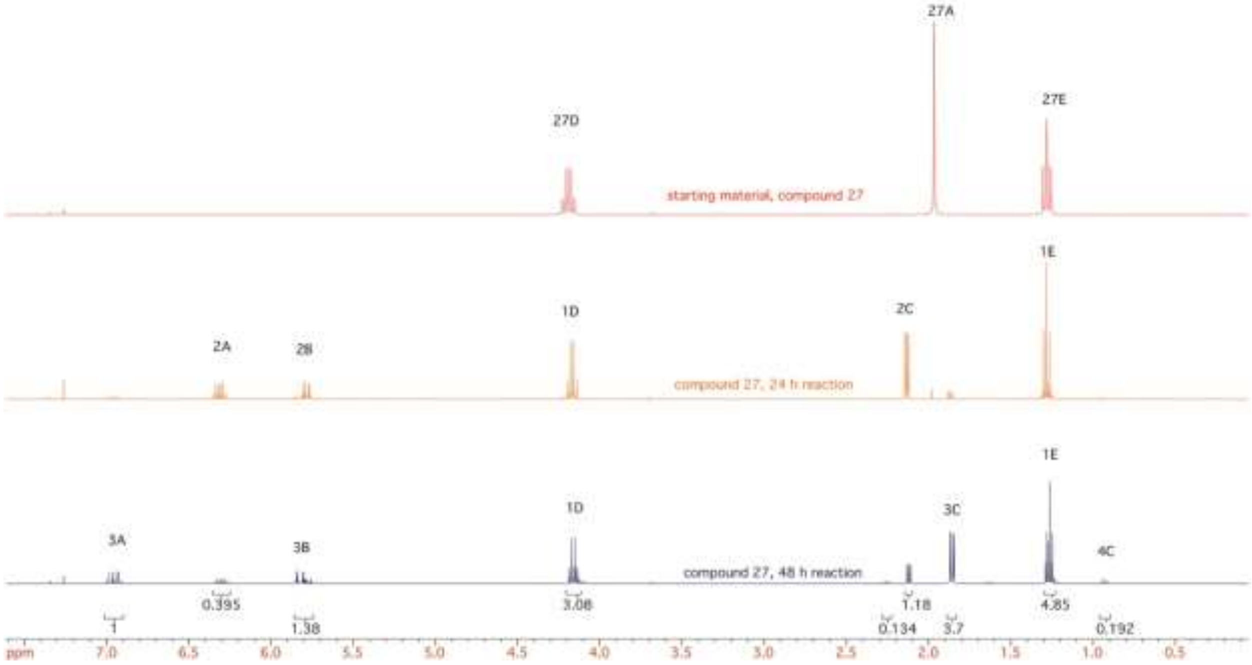

Figure S12. The ^{1}H NMR of compound **27** (top), after the 24 hour reaction (middle), and after the 48 hour reaction (bottom) with 5 mol% of C8 PdNP under 1 atm H_2 in $CDCl_3$.

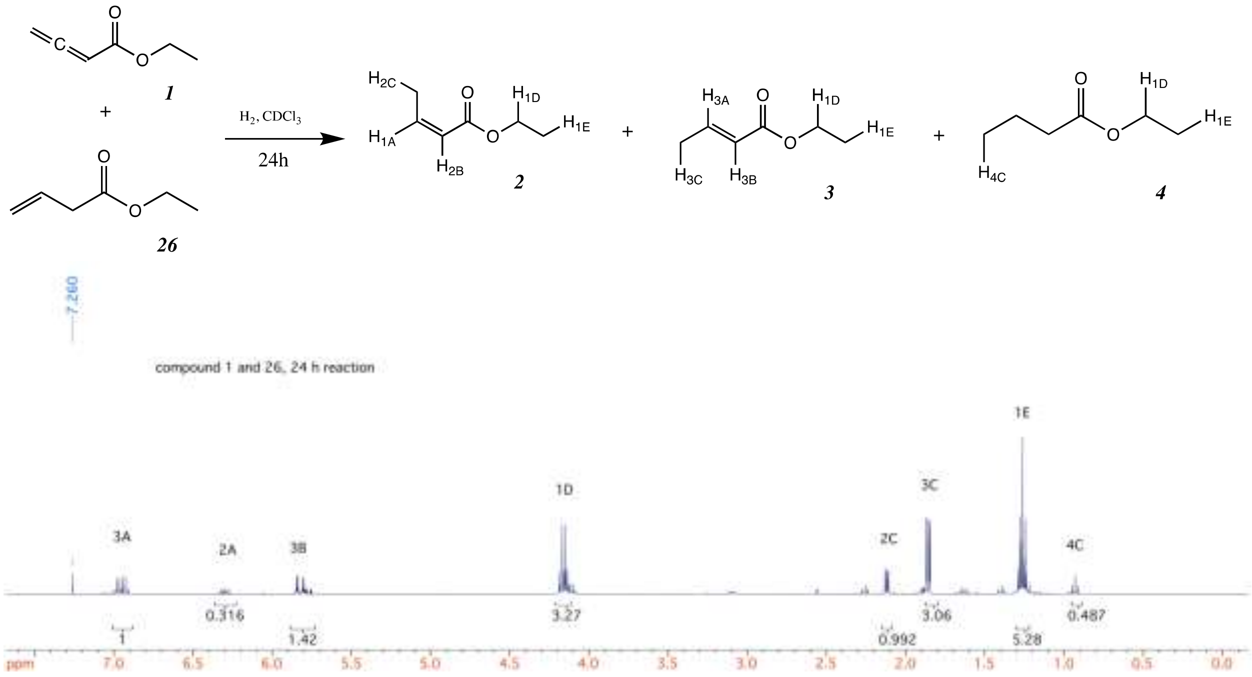

Figure S13. ^{1}H NMR of compound **1** and **26** dual-substrates after the 24 hour reaction with 5 mol% of C8 PdNP under 1 atm H_2 in $CDCl_3$.

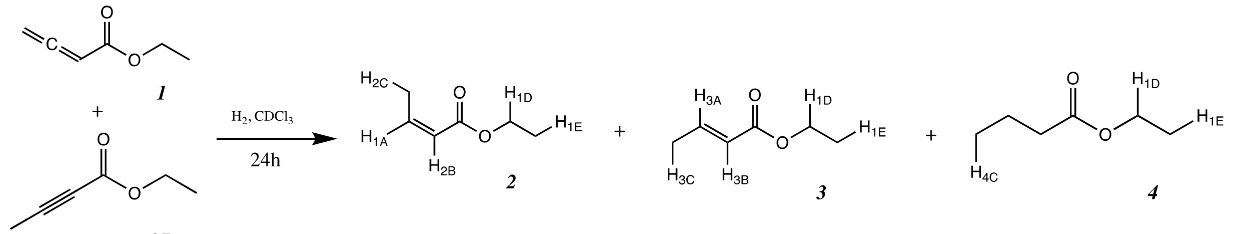

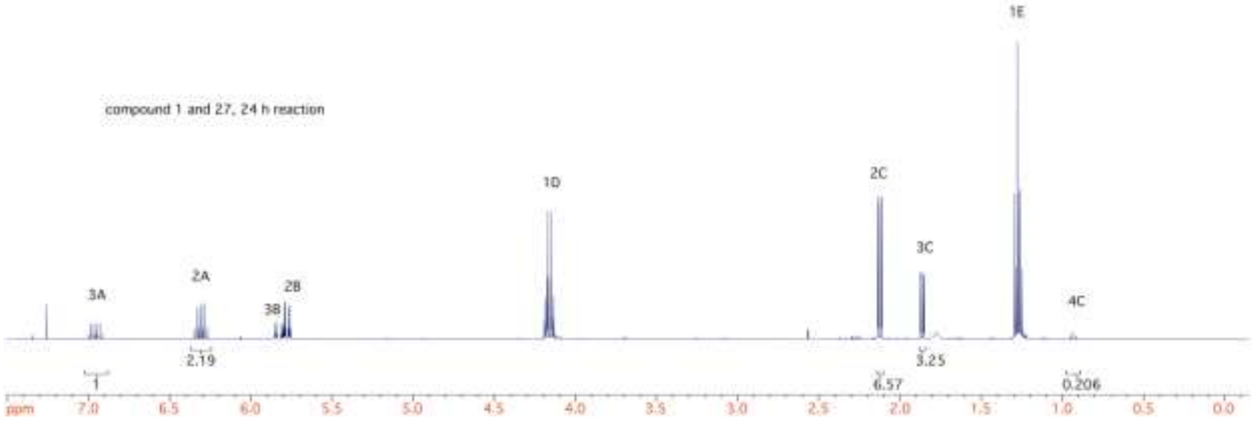

Figure S14. The ^{1}H NMR of compound **1** and **27** dual-substrates after the 24 hour reaction with 5 mol% of C8 PdNP under 1 atm H_2 in $CDCl_3$.
